# Tetraspanins as therapeutic targets in hematological malignancy: a concise review

**DOI:** 10.3389/fphys.2015.00091

**Published:** 2015-03-23

**Authors:** Kyle A. Beckwith, John C. Byrd, Natarajan Muthusamy

**Affiliations:** ^1^Division of Hematology, Department of Internal Medicine, The Ohio State UniversityColumbus, OH, USA; ^2^Division of Medicinal Chemistry, College of Pharmacy, The Ohio State UniversityColumbus, OH, USA; ^3^Department of Molecular Virology, Immunology, and Medical Genetics, The Ohio State UniversityColumbus, OH, USA

**Keywords:** tetraspanin, TSPAN, CD37, CD53

## Abstract

Tetraspanins belong to a family of transmembrane proteins which play a major role in the organization of the plasma membrane. While all immune cells express tetraspanins, most of these are present in a variety of other cell types. There are a select few, such as CD37 and CD53, which are restricted to hematopoietic lineages. Tetraspanins associate with numerous partners involved in a diverse set of biological processes, including cell activation, survival, proliferation, adhesion, and migration. The historical view has assigned them a scaffolding role, but recent discoveries suggest some tetraspanins can directly participate in signaling through interactions with cytoplasmic proteins. Given their potential roles in supporting tumor survival and immune evasion, an improved understanding of tetraspanin activity could prove clinically valuable. This review will focus on emerging data in the study of tetraspanins, advances in the clinical development of anti-CD37 therapeutics, and the future prospects of targeting tetraspanins in hematological malignancy.

## Introduction

Tetraspanins are transmembrane proteins which are ubiquitous among metazoans, with 33 family members identified in mice and humans (Maecker et al., 1997). The predominant view has been that tetraspanins are facilitators of signal transduction, providing organization to plasma membrane domains through lateral interaction with their numerous partners (Maecker et al., 1997; Hemler, 2005; Charrin et al., 2009). However, there is recent evidence that certain tetraspanins also recruit signaling proteins directly (Lapalombella et al., 2012). Tetraspanins have been reported to regulate diverse processes, including cellular migration, adhesion, activation, and apoptosis (Hemler, 2005). Furthermore, several tetraspanins influence cancer metastasis/progression and their functional roles in immune cells could impact anti-tumor immunity (Zoller, 2009; Veenbergen and Van Spriel, 2011; Hemler, 2014). This review will focus on tetraspanins expressed by immune cells and discuss therapeutic strategies targeting these proteins in hematological malignancies.

### Structure of tetraspanin proteins

**Figure 1:**
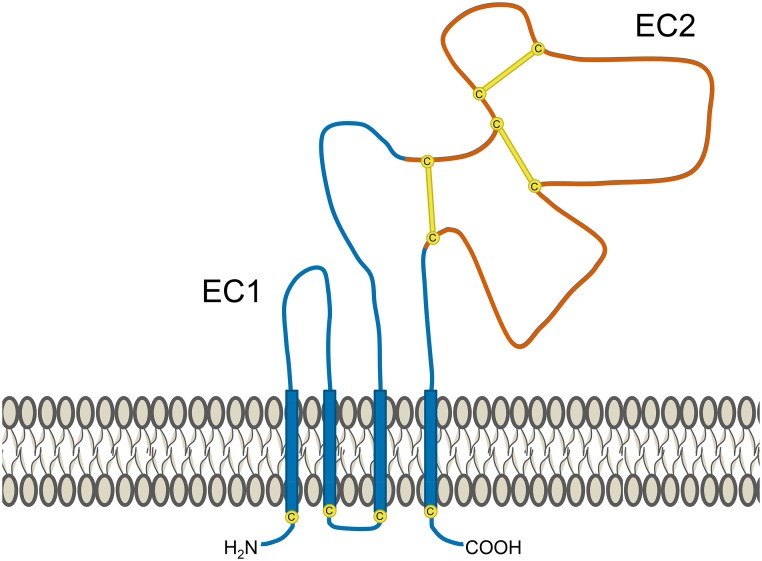
**Structural features of tetraspanins**. Several common features of tetraspanins are depicted here. They possess 4 transmembrane domains (which are highly conserved), two short cytoplasmic tails, and two extracellular portions known as the EC1 domain (small extracellular loop) and EC2 domain (large extracellular loop). Portions of the EC2 domain are conserved between various tetraspanins, but it also contains a highly variable region (shown in red). One of the features of this segment is the presence of 2–4 disulfide bonds (yellow lines) formed between cysteine residues (yellow circles), the number of which depend on the particular tetraspanin. The variable region of the EC2 domain contains binding sites for interactions with partner proteins and is frequently where epitopes for anti-tetraspanin antibodies are found. Many tetraspanins undergo palmitoylation at cysteine residues located near the intracellular border of the four transmembrane portions. Additionally, most tetraspanins also experience N-linked glycosylation at extracellular asparagine residues (not depicted).

Tetraspanins contain short N-terminal and C-terminal cytoplasmic tails, a small extracellular loop (EC1 domain), a large extracellular loop (EC2 domain) and four transmembrane domains (Figure 1). The EC2 domain contains a region conserved among tetraspanins, but also a highly variable region that is frequently involved in the specific interactions between tetraspanins and various non-tetraspanin partners (Yauch et al., 2000; Charrin et al., 2001; Shoham et al., 2006; Zevian et al., 2011). It is typical for tetraspanins to undergo extensive post-translational modification. Covalent attachment of palmitate to intracellular cysteine residues is implicated in mediating tetraspanin-tetraspanin interactions and assembly of tetraspanin-enriched domains that can support signaling (Berditchevski et al., 2002; Charrin et al., 2002; Yang et al., 2002, 2004). Furthermore, nearly all tetraspanins display extensive N-linked glycosylation at extracellular sites (Maecker et al., 1997). This glycosylation is likely to have functional relevance, as shown with CD82 and CD9, which could only influence motility or apoptosis when glycosylated (Ono et al., 1999, 2000). A variety of glycosylation patterns are observed across cell lines, including those of the same lineage, but it remains unknown whether these differences have any impact on tetraspanin function (Schwartz-Albiez et al., 1988; White et al., 1998).

### Tetraspanin interactions

Numerous *cis*-interactions occur between tetraspanins and neighboring plasma membrane proteins within what are known as tetraspanin-enriched microdomains (Hemler, 2005). These microdomains may function as signaling platforms, similar to lipid rafts but generally comprised of distinct components (Claas et al., 2001; Le Naour et al., 2006a; Mattila et al., 2013; Zuidscherwoude et al., 2014). Many tetraspanin interactions depend on binding to the extracellular EC2 domain (Yauch et al., 2000; Charrin et al., 2001; Shoham et al., 2006; Zevian et al., 2011), although transmembrane domains are also frequently involved (Charrin et al., 2001, 2003; Shoham et al., 2006). A diverse set of proteins interact with tetraspanins, including adhesion molecules (e.g., integrins), various immunoreceptors, and several intracellular signaling molecules. Table 1 provides a summary of proteins reported to interact with hematopoietic-restricted tetraspanins. A subset of interactions are discussed here, but a number of excellent reviews cover this topic in greater depth for additional tetraspanins (Tarrant et al., 2003; Charrin et al., 2009). Although numerous associations have been documented with other transmembrane proteins, there are fewer examples of tetraspanins interacting with cytoplasmic proteins. This is not surprising, given their short cytoplasmic tails are generally less than 20 amino acids in length (Maecker et al., 1997). This has contributed to the thought that tetraspanins do not directly participate in signal transduction. However, several tetraspanins have been reported to associate with intracellular signaling proteins. Common cytoplasmic partners include PI4K and PKC (Berditchevski et al., 1997; Zhang et al., 2001; Andre et al., 2006), but tetraspanins have also been shown to interact with several other signaling proteins (Clark et al., 2004; Little et al., 2004; Andre et al., 2006; Le Naour et al., 2006b; Lapalombella et al., 2012). While tetraspanins clearly influence signaling, it remains possible that some of these interactions could be indirect as a result of association with adapter proteins.

**Table 1:** **Proteins associated with hematopoietic-specific tetraspanins**.

**Tetraspanin**	**Interactions**	**References**
CD37	Syk, Lyn, SHP1, PI3Kδ, PI3Kγ	Lapalombella et al., 2012
	MHC-II	Angelisová et al., 1994
	Dectin-1	Meyer-Wentrup et al., 2007
CD53	α_4_β_1_ integrin	Mannion et al., 1996
	PKC	Zhang et al., 2001
	CD2	Bell et al., 1992
	CD20, MHC-I	Szöllósi et al., 1996
	MHC-II	Angelisová et al., 1994
	Unknown tyrosine phosphatase	Carmo and Wright, 1995
Tssc6	Glycoprotein IIb/IIIa (α_2b_β_3_ integrin)	Goschnick et al., 2006
TSPAN33	ADAM10	Haining et al., 2012

Tetraspanins have been largely discounted as potential cell-surface receptors on the basis of their structure, which protrudes at most 5 nm into extracellular space (Kitadokoro et al., 2001; Min et al., 2006). While their interactions do primarily occur in *cis*, recent publications have challenged the notion that tetraspanins cannot also function as receptors. CD9 is reported to have multiple soluble ligands, both acting as an alternative IL-16 receptor in mast cells (Qi et al., 2006) and binding PSG17, a placental protein released during pregnancy that can induce macrophages to release IL-10, IL-6, and TGFβ (Waterhouse et al., 2002). CD81 has been identified as an essential receptor for Hepatitis C virus (Pileri et al., 1998). CD82 has been described as a receptor for the endothelial cell-surface protein DARC. While expression of CD82 typically decreases tumor metastasis, this suppression is eliminated in DARC knockout mice (Bandyopadhyay et al., 2006). Despite these occasional reports, the capability of tetraspanins to bind endogenous ligands in a *trans*-fashion remains somewhat controversial in the field.

Anti-tetraspanin antibodies typically induce functional effects of a degree exceeding that observed in knockout mice, which often exhibit mild phenotypes (Levy et al., 1998), This may result from perturbation of tetraspanin-enriched microdomains, given the extensive network of proteins that interact with tetraspanins and their partners. The ability to directly influence a multitude of biological processes is rather unique compared to antibodies targeting most other surface proteins. While a potentially beneficial trait if it can be exploited, this also means that anti-tetraspanin therapeutics could have complex effects. For example, anti-CD9 antibodies decrease CXCR4-dependent transendothelial migration, but also increase adhesion to fibronectin, endothelial cells, and bone marrow stromal cells (Masellis-Smith and Shaw, 1994; Leung et al., 2011). To further complicate matters, antibodies targeting different epitopes may have distinct effects. Anti-CD9 antibody targeting a different epitope does not alter adhesion to fibronectin (a ligand of α_4_β_1_ integrin), instead increasing adhesion to laminin, an α_6_β_1_ integrin ligand (Gutierrez-Lopez et al., 2003). We will further explore the topic of tetraspanin-directed therapeutic strategies in a later section of this review, following a discussion of the potential tetraspanin targets expressed within the immune system.

## Tetraspanins in the hematopoietic system

Many of the tetraspanins present on immune cells are also found in a variety of other tissues, but some display hematopoietic-restricted expression, including CD37, CD53, Tssc6 (TSPAN32), and TSPAN33 (Tarrant et al., 2003; Heikens et al., 2007). The expression patterns of these tetraspanins are summarized in Table 2. Here we will individually discuss several tetraspanins present in normal and malignant cells, beginning with the four proteins primarily found in the hematopoietic system. Functional roles of the hematopoietic-restricted tetraspanins are also summarized in Table 3.

**Table 2:** **Expression pattern of tetraspanins in the hematopoietic system**.

**Tetraspanin**	**Expression**
CD37	B-cells (predominantly), T-cells, granulocytes, MO, DCs
CD53	B-cells, T-cells, granulocytes, MO, DC, NK cells, HSCs/HPCs
Tssc6	B-cells, T-cells, granulocytes, MO, DCs, platelets, erythroid cells, HPCs
TSPAN33	B-cells (activated), erythroid precursors, kidney (PCT, DCT, CD)
CD9	B-cells, T-cells, granulocytes, MO, DCs, platelets/megakaryocytes, HSCs/HPCs, various non-hematopoietic tissues
CD81	B-cells, T-cells, MO, DCs, NK cells, HPCs, various non-hematopoietic tissues
CD82	B-cells, T-cells, granulocytes, MO, DCs, HPCs, various non-hematopoietic tissues
CD151	B-cells, T-cells, neutrophils, MO, DCs, platelets/megakaryocytes, various non-hematopoietic tissues

*MO, monocytes/macrophages; DC, dendritic cell; HSC/HPC, hematopoietic stem cell/progenitor cell; PCT/DCT, proximal or distal convoluted tubules; CD, collecting ducts*.

*CD37, expression determined at protein level in human cells (Deckert et al., 2013) and by mRNA in mice (Van Spriel et al., 2009). CD53, expression by protein in humans (Olweus et al., 1993; Mollinedo et al., 1997; Barrena et al., 2005; Beckmann et al., 2007). Tssc6, expression determined at mRNA level in mice (Nicholson et al., 2000; Robb et al., 2001; Goschnick et al., 2006). TSPAN33, expression determined at both protein/mRNA level in humans (Luu et al., 2013) and by mRNA levels in mice (Heikens et al., 2007). Broadly expressed tetraspanins (CD9, CD81, CD82 CD151), expression at protein level (reviewed by Maecker et al., 1997; Tarrant et al., 2003; Zoller, 2009)*.

**Table 3:** **Function of hematopoietic-restricted tetraspanins**.

**Tetraspanin**	**Reported functions**
CD37	B-cell immunity: (1) Promotes T-cell dependent B-cell responses by mediating α_4_β_1_ integrin signaling in B-cells to support survival of IgG1-secreting cells (Knobeloch et al., 2000; Van Spriel et al., 2012) (2) Negatively regulates IgA production by B-cells (Van Spriel et al., 2009) a. In macrophages, CD37 negatively regulates fungal-induced Dectin-1 stimulation (and subsequent IL-6 production), which may contribute to increased IgA production (Meyer-Wentrup et al., 2007) T-cell immunity: (1) Complex role, but its involvement in DC migration makes CD37 essential for normal T-cell responses (Gartlan et al., 2013). However, it also… a. negatively regulates TCR signaling *in vitro* (Van Spriel et al., 2004) b. negatively regulates peptide/MHC presentation (Sheng et al., 2009)
CD53	Potential role in regulation of TNFα production (Bos et al., 2010)
	May promote cell survival (Voehringer et al., 2000; Yunta and Lazo, 2003)
Tssc6	Negatively regulates TCR signaling (Tarrant et al., 2002)
	Important for normal T-cell responses *in vivo*, yet appears to negatively regulate T-cell activation *in vitro* similar to CD37 (Gartlan et al., 2010)
	Platelet aggregation, by controlling GPIIb/IIIa signaling (Goschnick et al., 2006)
TSPAN33	Unknown role in erythropoiesis (Heikens et al., 2007; Haining et al., 2012)

*DC, dendritic cell; TCR, T-cell receptor; GPIIb/IIIa, glycoprotein IIb/IIIa*.

### CD37

This protein is most highly expressed by mature B-cells, although other immune cells express CD37 to a lesser degree (Link et al., 1986; Van Spriel et al., 2009; Deckert et al., 2013). It is absent in the earliest stages of B-cell development and is lost again following differentiation into plasma cells; a pattern mirrored by B-cells malignancies originating from various developmental stages (Barrena et al., 2005). CD37 is highly expressed in mature B-cell malignancies, such as non-Hodgkin lymphoma and chronic lymphocytic leukemia (CLL), but is low or absent in acute lymphoblastic leukemia and multiple myeloma. The expression pattern of CD37 has led to considerable interest in targeting this tetraspanin therapeutically (Zhao et al., 2007; Heider et al., 2011; Krause et al., 2012; Dahle et al., 2013; Deckert et al., 2013; Beckwith et al., 2014). This subject will be discussed in detail within a later section of the review.

**Figure 2:**
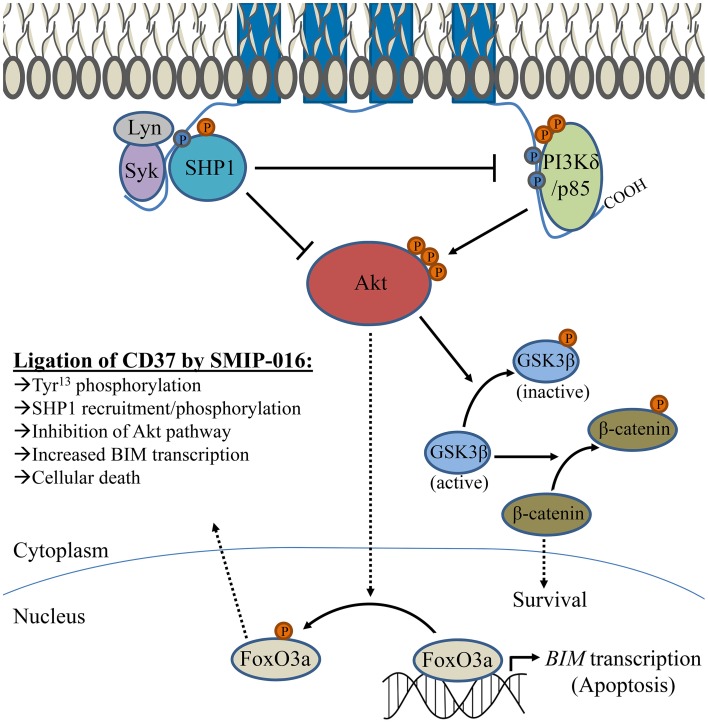
**Signaling pathway associated with CD37 ligation by SMIP-016**. Lapalombella et al. described a number of cytoplasmic proteins which can associate with CD37, as depicted in the diagram. Ligation by SMIP-016 leads to phosphorylation Tyr^13^ within an ITIM-like motif found in the N-terminal cytoplasmic tail, which associates with a complex of proteins that includes Syk, Lyn, and SHP1 (which likewise become phosphorylated). In addition, SMIP-016 induces phosphorylation of an ITAM-like motif (containing Tyr^274^ and Tyr^280^) located in the C-terminal cytoplasmic tail that recruits PI3Kδ. Mutational studies suggest that the events requiring the N-terminal ITIM drive apoptosis, while the C-terminal tail has a role in promoting cell survival. The proposed mechanism of anti-CD37 induced cellular death involves a balance between these signals, with preferential SHP1 activation driving apoptosis. SHP1 is capable of inactivating both PI3K and Akt. SMIP-016 decreases the nuclear localization of Akt, preventing phosphorylation of FoxO3a (and promoting retention in the nucleus) to allow transcription of pro-apoptotic BIM. An opposing signal is transduced through PI3Kδ recruited to the C-terminal ITAM, activating Akt and resulting in the downstream phosphorylation of GSK3β (which permits nuclear translocation of pro-survival β-catenin). However, the contribution of PI3Kδ to survival can be eliminated by either combination with a PI3K inhibitor or deletion of the ITAM-containing C-terminal domain of CD37. While both pro-survival and pro-apoptotic signaling pathways that are activated upon ligation by anti-CD37 SMIP-016, those that promote cellular death predominate. Several other CD37-targeted antibodies directly induce leukemia cell death, presumably in a similar fashion as SMIP-016/TRU-016. However, they do not require additional receptor crosslinking (by use of anti-Fc antibody to amplify the signal) as was observed with SMIP-016.

CD37-deficient mice exhibit defective IgG1 production in response to T-cell dependent antigens (Knobeloch et al., 2000), which is a consequence of decreased survival among IgG1-secreting B-cells in the days following antigen exposure (Van Spriel et al., 2012). It was demonstrated that CD37 has an important role in clustering α_4_β_1_ integrin (also known as VLA-4) on the plasma membrane. Absence of CD37 impaired integrin-dependent Akt signaling that is typically activated through interaction with follicular dendritic cells expressing a ligand of α_4_β_1_ integrin (Van Spriel et al., 2012). Similarly, ligation of CD37 by the antibody-derived peptide SMIP-016 also modulates the Akt pathway in B-cells (Lapalombella et al., 2012). However, SMIP-016 induces both pro-apoptotic Akt inactivation and opposing pro-survival phosphoinositide 3-kinase δ (PI3Kδ) activation. Analysis of its sequence suggested that the cytoplasmic tails of CD37 contained weak ITIM and ITAM-like motifs. Mutational studies support this function, providing evidence that the N-terminal ITIM can recruit SHP1 (which is capable of dephosphorylating/inactivating Akt) and the C-terminal ITAM can recruit PI3Kδ. While pro-survival and pro-apoptotic pathways are simultaneously induced by CD37 ligation, cellular death is favored. This is associated with increased BIM, a BH3-only Bcl-2 family protein that is critically important for its role in controlling mitochondrial-induced apoptosis. Figure 2 displays the signaling pathway implicated by the mechanistic studies of Lapalombella et al., which explains why several anti-CD37 therapeutics drive apoptosis in leukemia cells (Zhao et al., 2007; Heider et al., 2011; Krause et al., 2012; Lapalombella et al., 2012; Deckert et al., 2013; Beckwith et al., 2014). It is unknown why recruitment/activation of SHP1 (which promotes cellular death) is favored over that of PI3Kδ, although binding of anti-CD37 antibodies could cause conformational changes that alter how CD37 interacts with its partners. It should be noted that all of these therapeutic antibodies target the same epitope on CD37, thus distinct effects may be observed with antibodies directed at a different epitope. Apoptosis induction of this degree is unprecedented among anti-tetraspanin antibodies, raising questions as to whether cellular death is directly attributable to CD37 function or if there is an alternative explanation (Hemler, 2014). Using a secondary anti-Fc antibody to crosslink CD37/SMIP complexes could drastically alter organization of the tetraspanin microdomain. In addition, co-ligation of inhibitory FcγRIIb could contribute to apoptosis similar to how the effects of anti-CD9 antibodies in platelets were not CD9-driven but instead resulted from Fcγ receptor engagement (Worthington et al., 1990). While the impact of Fcγ receptor engagement cannot be completely ruled out, particularly in regard to cytotoxicity in CLL B-cells which express FcγRIIb, the pre-B 697 cell line used for mutant CD37 studies is unlike mature B-cells in that it lacks FcγRII (Suzuki et al., 2002; Lapalombella et al., 2012). Furthermore, newer CD37-targeted antibodies induce leukemia cell apoptosis without the need for a secondary anti-Fc crosslinker (Krause et al., 2012; Beckwith et al., 2014).

While the expression of CD37 is low in non-B cells (Van Spriel et al., 2009; Deckert et al., 2013), it still has important functions in T-cells, dendritic cells, and macrophages (Van Spriel et al., 2004; Meyer-Wentrup et al., 2007; Sheng et al., 2009; Gartlan et al., 2010, 2013). CD37 associates with Dectin-1 and appears to negatively regulate its activity in anti-fungal response, as IL-6 production is dramatically increased in CD37^−/−^ macrophages following Dectin-1 stimulation (Meyer-Wentrup et al., 2007). It is possible that this regulation is accomplished through recruitment of phosphatases by CD37, which can associate with its N-terminal domain (Lapalombella et al., 2012). IL-6 production by CD37^−/−^ cells likely supports the generation of IgA-secreting plasma cells, leading to excessive IgA secretion that ultimately provides these mice with resistance to fungal infections (Van Spriel et al., 2009). CD37 plays a complex role in T-cell responses, as made evident by the seemingly contradictory results of *in vitro* and *in vivo* studies. *In vitro*, CD37^−/−^ dendritic cells are hyperstimulatory toward T-cells, and the data imply that CD37 negatively regulates peptide-MHC presentation (Sheng et al., 2009; Gartlan et al., 2010). Furthermore, CD37 in T-cells may play a negative regulatory role in T-cell receptor (TCR) signaling. CD37-deficient T-cells proliferate more rapidly in response to TCR stimulation, which could be a result of decreased Lck phosphorylation (Van Spriel et al., 2004). While the *in vitro* data imply a negative regulatory role for CD37 in T-cell responses, the opposite is observed using *in vivo* models. Mice deficient for CD37 are more susceptible to infection with murine malaria (Gartlan et al., 2010) and fail to reject syngeneic tumor cells transfected to express a foreign antigen (Gartlan et al., 2013). These discrepancies are explained by the observation that dendritic cells from CD37^−/−^ mice have impaired migratory and adhesion capabilities, which clearly overshadows other potential contributions of CD37 (Gartlan et al., 2013). It remains unclear whether the hyperproliferative phenotype of CD37-deficient T-cells is relevant beyond *in vitro* studies, but providing CD37^−/−^ mice with wildtype dendritic cells did not appear to significantly increase the number of IFNγ producing T-cells relative to wildtype mice.

### CD53

The tetraspanin CD53 is expressed by virtually all immune cells (Tarrant et al., 2003), a subset of hematopoietic stem cells (Beckmann et al., 2007), and in a variety of hematological malignancies (Barrena et al., 2005). CD53 mRNA transcripts increase in response to stimulation (Amiot, 1990; Mollinedo et al., 1998), although its protein levels decrease in neutrophils despite having increased transcript, so this data should be interpreted carefully (Mollinedo et al., 1998). There is substantial evidence that CD53 has an important role in the immune system. In humans, CD53 deficiency is associated with recurrent candida, intestinal, and upper respiratory tract infections (Mollinedo et al., 1997). With this clinical study it is unclear whether the altered CD53 expression resulted from mutation of the gene itself or a more complex regulatory defect, but it was reported to be decreased or absent in multiple cell types. In another study, a single nucleotide polymorphism in the CD53 gene strongly correlated with serum TNFα, suggesting this tetraspanin could have some role in mediating cytokine production (Bos et al., 2010). Furthermore, it has been implicated in the regulation of apoptosis by several studies. Elevated CD53 transcript was observed in radiation-resistant lymphoma cell lines (Voehringer et al., 2000). In addition, ligation of CD53 by antibody increased Akt phosphorylation and protected lymphoid tumor cell lines from death while under conditions of serum starvation (Yunta and Lazo, 2003). CD53 also associates with PKC (Zhang et al., 2001), which becomes activated following treatment with anti-CD53 antibody (Bosca and Lazo, 1994). With all anti-tetraspanin antibodies, however, conclusions about function should be made cautiously as their effects could be either agonistic or antagonistic.

### Tssc6 (TSPAN32)

The expression of Tssc6 mRNA is observed in hematopoetic progenitors, B-cells, T-cells, myeloid cells, and erythroid cells (Nicholson et al., 2000). What little we know of its function has been learned from the knockout mouse model (Tarrant et al., 2002). Despite being expressed widely among cells of hematopoetic origin, few phenotypic changes were observed in Tssc6^−/−^ mice. There were no defects in hematopoietic cell development (erythroid, lymphoid, or myeloid), response by neutrophils to acute infection was normal, and immunoglobulin production at baseline or after immune challenge was unaltered. Similar to CD37^−/−^ T-cells, however, Tssc6^−/−^ T-cells exhibit increased proliferation in response to TCR stimulation and dendritic cells are hyperstimulatory to T-cells (Tarrant et al., 2002; Gartlan et al., 2010). Tssc6^−/−^ mice also have poor CD8+ responses during infection, which is significantly worse in CD37^−/−^Tssc6^−/−^ mice (Gartlan et al., 2010). This discrepancy between *in vitro* and *in vivo* data has not yet been addressed as it has been in CD37^−/−^ mice, but it would be unsurprising if migratory/adhesion defects were similarly involved given that tetraspanins commonly have a role in mediating these activities. While evident that these tetraspanins have certain complimentary functions, it should also be noted that Tssc6^−/−^ mice produce immunoglobulins normally (and the CD37^−/−^ phenotype is not more severe in CD37^−/−^Tssc6^−/−^ mice), and thus they also possess unique roles in the immune system.

### TSPAN33

The final hematopoietic-restricted tetraspanin to be discussed in this review, TSPAN33, was originally described in erythroid precursors (Heikens et al., 2007; Haining et al., 2012). Interestingly, TSPAN33 maps to a hotspot for deletions in acute myeloid leukemia and myelodysplastic syndrome, although the significance of this is unknown (Chen et al., 2005). Knockout of the gene coding for TSPAN33, also called Penumbra, led to anemia to approximately 30% of mice between 6 and 17 months of age (Heikens et al., 2007). While young mice were not anemic, they did display an increase in erythrocytes with a “target cell” appearance, perhaps indicative of structural defects that could increase their rate of destruction. Splenomegaly was noted to be more common, even in non-anemic mice, and was accompanied by an increase in splenic erythrocytes. It is plausible that the splenomegaly was due to extramedullary hematopoiesis occurring as a compensatory mechanism in response to erythrocyte loss. TSPAN33 was later described as interacting with ADAM10, a metalloprotease involved in cell maturation that could potentially influence erythrocyte development in TSPAN33^−/−^ mice (Haining et al., 2012).

A recent publication challenges the notion that TSPAN33 is primarily expressed in erythroid precursors (Luu et al., 2013). The authors report it is highest (by mRNA and protein) in activated B-cells, and is comparatively low in human bone marrow and absent in other lineages of activated leukocytes. Consistent with this, TSPAN33 protein was also observed in Burkitt lymphoma cell lines and was uniformly expressed in diffuse large B-cell lymphoma biopsies. Elevated TSPAN33 transcripts were also detected in systemic lupus erythematosus and rheumatoid arthritis patient samples, pathological states where activated B-cells are expected to be present. In addition, this tetraspanin appears to be expressed in the kidney (proximal/distal convoluted tubules and collection ducts), albeit at lower levels than activated B-cells. Although this would mean that TSPAN33 is not entirely restricted to hematopoietic cells in humans, its specificity would remain higher than most tetraspanins and it may still have utility as a therapeutic target. While the expression of TSPAN33 was low in bone marrow, the authors did not examine cells of the erythrocyte lineage in isolation. Deposited microarray data agrees with the original reports that TSPAN33 is highly expressed in mouse erythroid precursors, but this pattern does not appear to be mirrored in human bone marrow (Seita et al., 2012). This could partly explain the disagreement between these studies. Overall, these contradictory results are intriguing but will require further confirmation. The introduction of improved, validated antibodies for studying TSPAN33 will certainly help, as the quality of currently available reagents is underwhelming.

### Other tetraspanins

A number of additional tetraspanins are functionally relevant to immune cells. However, they are expressed in many different tissues, thus have reduced utility as therapeutic targets in hematological malignancy. Therefore, we will only briefly discuss a small subset of these proteins. In particular, CD9, CD81, CD82, and CD151 have known roles in the immune system. It is well appreciated that CD81 interacts with CD19 and acts as a co-receptor in B-cell receptor signaling, but it appears to have roles in T-cells as well (Maecker and Levy, 1997; Levy, 2014). Antibodies targeting either CD81 or CD9 have both been reported to deliver co-stimulatory signals to T-cells in a CD28-independent manner (Tai et al., 1996; Witherden et al., 2000). CD81 and CD82 have been shown to associate with one another, CD4, and CD8 in T cells (Imai et al., 1995). In B-cells, CD82 has also been shown to associate with CD19 (Horváth et al., 1998).

The prognostic significance of CD9 expression varies between different types of cancer. Low expression in solid tumors is sometimes associated with poor prognosis, while in other cases the opposite is true (Romanska and Berditchevski, 2011). In hematological malignancy, CD9 expression has been studied in multiple myeloma and monoclonal gammopathy of unknown significance (MGUS), which precedes myeloma development. Barrena et al. observed that CD9 expression was higher in samples from patients with MGUS (Barrena et al., 2005). A later retrospective study investigated CD9 expression in bone marrow aspirates from 81 myeloma patients by flow cytometry and discovered that more patients with inactive disease expressed CD9 (60.7%) than those with active disease (33.9%) (De Bruyne et al., 2008). Absence of CD9 expression at diagnosis also correlated with decreased survival. Interestingly, the transfection of myeloma cell lines with CD9 was reported to increase their susceptibility to lysis by NK and T-cells (Shallal and Kornbluth, 2000), offering a potential explanation for how CD9 downregulation could benefit tumor cells. Transfection of myeloma cell lines with CD9 also increases their sensitivity to the proteosome inhibitor bortezomib, which is frequently used in the treatment of myeloma, suggesting that loss of CD9 can influence drug resistance (Hu et al., 2014).

CD151 is known to interact with α_3_β_1_ integrin, through which this tetraspanin has been reported to mediate neutrophil motility (Yauch et al., 1998). Similar to CD37 and Tssc6, CD151 is also implicated in the negative regulation of T-cell activation. CD151^−/−^ murine T-cells are hyperproliferative in response to TCR-stimulation (Wright et al., 2004). Likewise, CD151^−/−^ dendritic cells are hyperstimulatory to T-cells, although this appears to be through regulation of co-stimulation rather than influencing MHC/peptide presentation as CD37 does (Sheng et al., 2009). However, conclusions from these *in vitro* data should be made carefully, given that T-cells from CD37^−/−^ and Tssc6^−/−^ mice have similar phenotypes, yet poor T-cell responses are observed *in vivo* (Van Spriel et al., 2004; Sheng et al., 2009; Gartlan et al., 2010, 2013).

## Targeting tetraspanins in hematological malignancy

Antibody-based strategies for treating cancer have rapidly increased in prevalence since anti-CD20 rituximab was introduced to the clinic. More than a dozen antibodies have been approved by the U.S. Food and Drug Administration (FDA) for cancer therapy and hundreds of ongoing human trials are registered at clinicaltrials.gov (Scott et al., 2012). The first attempts to develop a tetraspanin-targeted therapy predate the approval of rituximab by nearly a decade, when ^131^I –labeled murine anti-CD37 antibody was tested in a small cohort of non-Hodgkin lymphoma (NHL) patients (Press et al., 1989). While the early results were promising, targeting CD20 was quickly becoming the favored approach and CD37 was subsequently neglected for many years. Anti-CD37 therapy has experienced a recent resurgence, with five different targeting approaches being explored in B-cell malignancies. While several tetraspanins may be promising targets for cancer therapy, CD37 is by far the furthest in terms of clinical development (as summarized in Table 4). Thus, we will begin by reviewing advances in anti-CD37 therapy, to be followed by a broader discussion of tetraspanin-targeted therapy in hematological malignancy.

**Table 4:** **Clinical development of anti-CD37 therapies**.

**Therapeutic**	**Type**	**Clinical trials**	**Description**
Otlertuzumab (TRU-016)	mAb-derived polypeptide	A. Phase 1/1b (NCT00614042)	A. Treatment naïve/relapsed CLL (Byrd et al., 2014) and relapsed NHL (Pagel et al., 2015)
		B. Phase 1/2 (NCT01188681)	B. TRU-016 + bendamustine vs. bendamustine in relapsed CLL (Robak et al., 2013)
		C. Phase 1b (NCT01644253)	C. TRU-016 + rituximab in treatment naïve CLL
		D. Phase 1 (NCT01317901)	D. TRU-016 + rituximab + bendamustine in relapsed NHL (Gopal et al., 2014)
BI 836826 (mAb 37.1)	Fc-engineered IgG1	Phase 1	Details not yet available. Trials in both CLL and NHL are anticipated
IMGN529	antibody-drug conjugate	Phase 1 (NCT01534715)	NHL patients with relapsed/refractory disease
Betalutin	^177^Lu radioimmunotherapy	Phase 1/2	Relapsed NHL patients (Kolstad et al., 2014)

### Otlertuzumab (TRU-016)

**Figure 3:**
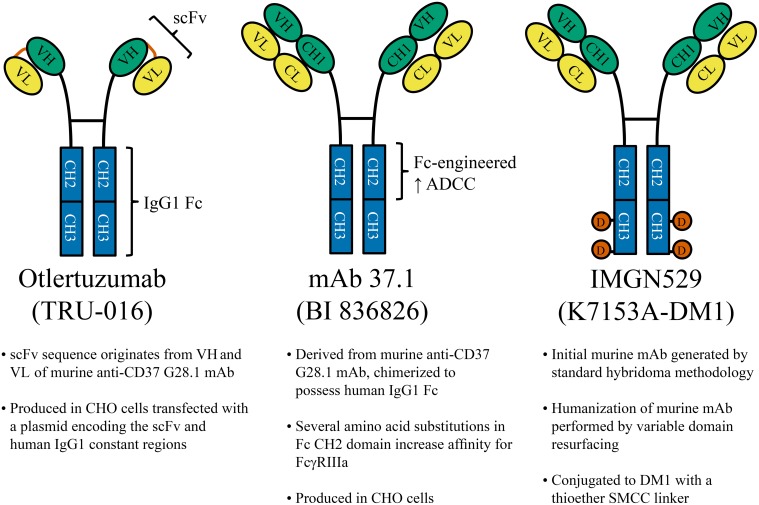
**CD37-Targeted antibody therapeutics**. Several anti-CD37 therapies under clinical development are shown. **Left:** Otlertuzumab is an ADAPTIR™ molecule, constructed from an anti-CD37 single-chain variable fragment (scFv; a binding domain formed by linking the heavy and light chain variable regions of an immunoglobulin) which has been fused to the hinge region and Fc domain of human IgG1. **Middle:** mAb 37.1 is an Fc-engineered IgG1 with specific amino acid substitutions within the Fc region to increase ADCC mediated by effectors such as NK cells and macrophages. **Right:** IMGN529 is a humanized anti-CD37 IgG1 (K7153A) conjugated to 3–4 molecules of cytotoxic drug (DM1) by stable thioether bonds. mAb, monoclonal antibody; CHO, Chinese hamster ovary; VH, heavy chain variable region; VL, light chain variable region; CH, heavy chain constant region (1, 2, or 3); CL, light chain constant region; D (orange circles), DM1; SMCC, N-succinimidyl-4-(N-maleimidomethyl)cyclohexane-1-carboxylate.

CD37 is highly expressed on the surface of human B-cells (where its antigen density is at least 15 times greater than on non-B leukocytes) and it is present in the vast majority of CLL and NHL cases (Deckert et al., 2013), making it an attractive target for immunotherapy. Several CD37-targeting antibody-based therapeutics have been developed which are currently being evaluated in the clinic (Zhao et al., 2007; Heider et al., 2011; Deckert et al., 2013). Otlertuzumab was the first of these to begin clinical trials. This therapeutic is a humanized, antibody-derived CD37-targeting peptide developed using the ADAPTIR™ platform. Mono-specific ADAPTIR molecules are built from a single-chain variable fragment (a binding domain formed by linking the heavy and light chain variable regions of an immunoglobulin), fused to the hinge region and Fc domain of human IgG1 (Figure 3). These molecules form antibody-like dimers that are smaller than IgG1 (intended to increase tissue penetration), but otherwise retain similar pharmacokinetics and activity as traditional IgG1. Preclinical studies using SMIP-016, a tool molecule not fully humanized (but containing human IgG1 Fc), demonstrated superior NK cell-mediated antibody dependent cellular cytotoxicity (ADCC) compared to anti-CD20 rituximab. This therapy also directly killed CLL tumor cells through induction of caspase-independent apoptosis when in the presence of anti-Fc crosslinker (Zhao et al., 2007). As discussed earlier, SMIP-016 was shown to induce both pro-apoptotic Akt inactivation and (to a lesser extent) pro-survival PI3Kδ activation (Lapalombella et al., 2012). The simultaneous activation of these opposing signaling pathways provides an opportunity to utilize unique combination strategies for anti-CD37 therapies. Indeed, Lapalombella et al. showed that SMIP-016 cytotoxicity against CLL B-cells was enhanced by the addition of either a pan-PI3K inhibitor (LY294002) or the PI3Kδ-selective CAL-101 (idelalisib; now FDA approved for CLL therapy). Further investigation of this potential combination is warranted, and should also be explored with newer anti-CD37 therapies that can more efficiently induce apoptosis without dependence on additional crosslinking (Heider et al., 2011; Deckert et al., 2013).

Otlertuzumab has been tested in CLL and NHL patients during recent clinical trials (Byrd et al., 2014; Gopal et al., 2014; Pagel et al., 2015). A phase I study in CLL observed modest single-agent activity and found it to be well tolerated (Byrd et al., 2014). Peripheral lymphocyte reduction was observed in 75.5% of patients with elevated initial lymphocyte counts, but overall response rate (ORR) was only 23% (19/83 patients) by NCI-96 criteria. Only partial responses (PR) were observed, which were more common among treatment-naïve CLL patients (6/7) or those who received 1 or 2 previous therapies (12/28). These results were encouraging, given that responses to single-agent rituximab are also limited (Byrd et al., 2001; Huhn et al., 2001), but can be dramatically improved by combination with chemotherapy (Keating et al., 2005). Similarly, an early report from a randomized Phase II trial in relapsed CLL demonstrates the improved efficacy of otlertuzumab when combined with bendamustine (NCT01188681). Patients receiving otlertuzumab plus bendamustine had an ORR of 80% (16/20 patients) with 20% achieving a complete remission (CR), while those treated with bendamustine alone had an ORR of only 42% (10/24) with a CR rate of 4% (Robak et al., 2013). A single-agent trial of otlertuzumab was also performed in NHL patients with follicular lymphoma, Waldenström's macroglobulinemia, or mantle cell lymphoma (Pagel et al., 2015). Responses were limited to 2 of 16 patients (12%). However, another phase I study evaluated the combination of otlertuzumab, rituximab, and bendamustine in 12 patients with indolent NHL (follicular, mantle cell, and small lymphocytic) who had relapsed after receiving treatment regimens which included rituximab (Gopal et al., 2014). Two doses of otlertuzumab were tested (10 or 20 mg/kg) with 6 patients per dose. This regimen was well tolerated and achieved an ORR of 83% (10/12), with four CRs. All of the patients receiving the higher dose (6/6) responded, with 2 CRs and 4 PRs. Overall, these clinical studies highlight the promise of anti-CD37 therapies, particularly in combination with other agents. The ongoing clinical evaluation of otlertuzumab and other CD37-targeted therapies is summarized in Table 4.

### Anti-CD37 therapeutics with enhanced ADCC

Several newer CD37-targeted therapeutics have the potential to surpass the clinical benefits observed with otlertuzumab. To mediate ADCC, IgG1 requires covalent attachment of oligosaccharides at Asn^297^ within its Fc region, but eliminating fucose from this carbohydrate structure is known to improve ADCC (Shinkawa et al., 2003). A non-fucosylated variant of otlertuzumab has been generated which has enhanced binding to FcγRIIIa, resulting in improved NK cell mediated ADCC and phagocytosis by macrophages (Rafiq et al., 2013). Alternatively, ADCC can be enhanced by mutating certain amino acid residues within the Fc region of IgG1 (Lazar et al., 2006). This approach was taken in the generation of mAb 37.1 (Figure 3), an IgG1 with specific mutations in the CH2 domain that augment ADCC (Heider et al., 2011; Krause et al., 2012). It is worth noting that this anti-CD37 antibody also directly induces leukemia cell apoptosis, but in contrast to otlertuzumab it does not require anti-Fc crosslinker. In addition, mAb 37.1 depleted B-cells in a human CD37 transgenic mouse model, although this was not in the context of malignant disease (Heider et al., 2011). Clinical trials evaluating the humanized version of this antibody are anticipated in both Europe and the United States.

### CD37-targeted delivery of cytotoxic agents

This final broad category of therapies utilizes anti-CD37 antibodies to guide cytotoxic agents to tumor cells. CD37 internalizes moderately faster than CD20 when bound by antibody (Press et al., 1994), yet not so quickly that ADCC is prevented (Zhao et al., 2007; Heider et al., 2011; Krause et al., 2012; Deckert et al., 2013; Beckwith et al., 2014). This affords an opportunity to exploit the unique properties of CD37 to generate therapeutics that: (1) maintain the Fc-mediated effector functions of IgG1, (2) deliver toxin into tumor cells through endocytosis, and (3) mediate potent antibody-induced apoptosis. The CD37-targeted antibody-drug conjugate IMGN529 has each of these functions, giving it a unique repertoire of mechanisms among therapeutics for B-cell malignancy (Deckert et al., 2013; Beckwith et al., 2014). IMGN529 is a humanized anti-CD37 IgG1 conjugated to DM1 (Figure 3), a drug which inhibits microtubule assembly during mitosis (Deckert et al., 2013). Unlike otlertuzumab (but analogous to mAb 37.1), CLL B-cells treated with the unconjugated antibody-component of IMGN529 undergo extensive apoptosis without the need for anti-Fc crosslinking antibody. Given that anti-CD37 antibodies do not react with mouse CD37, a transgenic mouse expressing human CD37 was generated and crossed with a commonly used model of CLL (Beckwith et al., 2014). In this model, IMGN529 rapidly depleted peripheral leukemia, eliminated the proliferative subset of tumor cells within lymphoid tissues, and improved overall survival. While these results are promising, it remains to be seen whether the additional delivery of anti-proliferative drug will be more effective in humans than other methods of targeting CD37, particularly in more indolent diseases like CLL. Currently, IMGN529 is being evaluated in NHL as part of an ongoing clinical trial (NCT01534715). This trial has encountered some early difficulties, with several patients experiencing grade III/IV neutropenia that can be largely avoided with the addition of corticosteroids and G-CSF (Stathis et al., 2014). While possible that the low level of CD37 expression on neutrophils results in their direct elimination, evidence obtained from mouse models is suggestive of cell redistribution (Deckert et al., 2014). Only relatively low doses of IMGN529 have been tested thus far, but 4 of 10 relapsed/refractory diffuse large B-cell lymphoma patients have responded to therapy (1 CR, 3 PR). This is expected to improve at higher doses, now that dose escalation is continuing with prophylaxis that addresses the neutropenia.

Similar to the above approach, anti-CD37 antibody can be used to deliver radioactive isotopes to tumor cells. This was first explored over 20 years ago (Press et al., 1989), but the renewed interest in targeting CD37 has lead to the reemergence of CD37-directed radioimmunotherapy (Dahle et al., 2013; Repetto-Llamazares et al., 2014). There are currently two FDA-approved radiolabeled antibodies that target CD20 (Scott et al., 2012). However, the propensity of CD37 to internalize may make it a superior target, given that this occurs 10 times faster with ^177^Lu conjugated to anti-CD37 tetulomab compared to anti-CD20 rituximab (Dahle et al., 2013). A phase I/II trial recently initiated in Europe is exploring this therapeutic strategy in NHL using Betalutin, a ^177^Lu-conjugated anti-CD37 antibody. Thus far, 7 of 11 patients on this trial have responded (ORR of 64%) with 4 CRs and 3 PRs (Kolstad et al., 2014).

A third approach to CD37-targeted drug delivery is the use of immunoliposomes coated in antibody (Yu et al., 2013). CD37-coated immunoliposomes effectively delivered cytotoxic drug to cell lines and CLL B-cells, and specificity could be altered by using a dual targeting approach with an additional CD19 or CD20 antibody. Furthermore, CD37 immunoliposomes that were not loaded with drug were capable of inducing apoptosis in CD37+ cells, presumably due to a crosslinking effect. While this approach is interesting experimentally, transitioning to full clinical development is quite complex due to formulation related issues.

### Future directions for tetraspanin-targeted therapy

Thus far, CD37 represents the only tetraspanin that has been targeted therapeutically in humans. Indeed, with greater than 15 times the antigen density on B-cells compared to other leukocytes (Deckert et al., 2013), it has a significant advantage over most tetraspanin targets which have expression in a variety of cell types. Antibodies targeting CD9 and CD151 have demonstrated promising activity in xenografts of solid tumors into mice (Zijlstra et al., 2008; Nakamoto et al., 2009). However, it is difficult to extrapolate these results to humans given the limitations of the models, which prevent assessment of toxicity. Specifically, these antibodies lack cross-reactivity with the equivalent mouse tetraspanins and the targeted antigens are expected to be highly expressed on a number of healthy cell types in patients. Even in tumors with increased expression of a tetraspanin, it may prove difficult to selectively target those cells. In the case of CD37, expression on non-B cells appears to be under the threshold needed for antibody-mediated killing, given the lack of cytotoxicity against other leukocytes in whole blood assays (Deckert et al., 2013; Beckwith et al., 2014) and the observation that T-cell numbers were unaltered in patients treated with otlertuzumab (Byrd et al., 2014). However, even low expression could have consequences in some contexts, as it may be responsible for the neutropenia observed in the IMGN529 trial (Stathis et al., 2014).

A potential alternative is to block the functions of more widely expressed tetraspanins with antibodies that lack effector functions or by disrupting interactions using soluble tetraspanin EC2 domains (Barreiro et al., 2005). However, it is unclear what kind of undesirable effects could result from doing so, given our still limited understanding of tetraspanin functions. Knockdown with siRNA has been successful in disrupting the activities of some tetraspanins (Barreiro et al., 2005), although in many cases it may do very little given that the phenotype of tetraspanin knockout mice is often mild in comparison to the effects induced by antibodies. If knockdown of a more widely expressed tetraspanin disrupts tumor function successfully, then perhaps the safest route of action is delivery of siRNA by immunoliposomes that are guided by antibodies targeting a more tumor-restricted antigen.

A number of tetraspanins could be functionally relevant in hematological malignancy given the numerous roles identified in normal immune cells. If possible, it would be advantageous for anti-tumor therapy to exploit antibody-mediated recruitment of effector cells or complement. However, a more limited approach (as discussed above), may be necessitated for even tetraspanins restricted to the hematopoietic system. For example, CD53 is widely expressed by many immune cells and even some hematopoietic stem cells (Table 2). Administering anti-CD53 IgG1 could lead to even more significant loss of non-tumor cells than CD52-targeting alemtuzumab, which is typically reserved for salvage therapy in CLL due to widespread CD52 expression among non-B leukocytes. It would be difficult to justify the pursuit of such a therapy given the number of more specific antibody therapeutics and small molecule inhibitors in clinical development. Therefore, even those tetraspanins with hematopoietic-restricted expression could demand an approach such as siRNA delivery, antibodies without effector functions, or other alternatives.

The recent report that TSPAN33 is highly expressed by activated B-cells and in several B-cell malignancies is intriguing (Luu et al., 2013), but additional studies are required to resolve conflicts with previously published work (Heikens et al., 2007; Haining et al., 2012). Surface expression of TSPAN33 on human erythroid precursors (and other cell types) should be more thoroughly investigated in human cells. Although some expression of TSPAN33 was observed in the kidney, it was not present in glomeruli and thus should be largely inaccessible to therapeutic antibodies (Luu et al., 2013). Therefore, TSPAN33 may represent a useful therapeutic target in B-cell malignancies (or autoimmune diseases) characterized by B-cells with an activated phenotype.

Can the relative ease by which CD37 is targeted by antibody therapeutics be replicated for other tetraspanins, or is it an anomaly? Their expression patterns may simply not be conducive to targeting with IgG1, and thus antibodies that do not recruit effector cells or other alternative approaches should be strongly considered. Overall, attempts to target widely expressed tetraspanins could present challenges, but it will become easier to develop safe and effective therapeutic strategies as we continue to better understand their functional roles in various malignancies and normal cell types.

### Conflict of interest statement

The authors declare that the research was conducted in the absence of any commercial or financial relationships that could be construed as a potential conflict of interest.
